# A genome-wide association study identifies three novel genetic markers for response to tamoxifen: A prospective multicenter study

**DOI:** 10.1371/journal.pone.0201606

**Published:** 2018-08-30

**Authors:** Hiroshi Onishi, Chihiro Udagawa, Michiaki Kubo, Seigo Nakamura, Sadako Akashi-Tanaka, Takashi Kuwayama, Chie Watanabe, Tomoko Takamaru, Hiroyuki Takei, Takashi Ishikawa, Kana Miyahara, Hiroshi Matsumoto, Yoshie Hasegawa, Yukihide Momozawa, Siew-Kee Low, Goro Kutomi, Hiroaki Shima, Fukino Satomi, Minoru Okazaki, Hisamitsu Zaha, Mai Onomura, Ayami Matsukata, Yasuaki Sagara, Shinichi Baba, Akimitsu Yamada, Kazuhiro Shimada, Daisuke Shimizu, Koichiro Tsugawa, Arata Shimo, Mikael Hartman, Ching-Wan Chan, Soo Chin Lee, Itaru Endo, Hitoshi Zembutsu

**Affiliations:** 1 Division of Genetics, National Cancer Center Research Institute, Tokyo, Japan; 2 Department of Gastroenterological Surgery, Yokohama City University Graduate School of Medicine, Yokohama, Japan; 3 RIKEN, Center for Integrative Medical Sciences, Yokohama, Japan; 4 Division of Breast Surgical Oncology, Department of Surgery, Showa University School of Medicine, Tokyo, Japan; 5 Department of Breast Surgery, Nippon Medical School, Tokyo, Japan; 6 Department of Breast Surgery, Tokyo Medical University, Tokyo, Japan; 7 Department of Breast Surgery, Saitama Cancer Center, Saitama, Japan; 8 Department of Breast Surgery, Hirosaki Municipal Hospital, Hirosaki, Japan; 9 1st Department of Surgery, Sapporo Medical University, Sapporo, Japan; 10 Department of Breast Surgery, Sapporo Breast Surgical Clinic, Sapporo, Japan; 11 Department of Breast Surgery, Nakagami Hospital, Okinawa, Japan; 12 Department of Breast Surgery, Sagara Hospital, Kagoshima, Japan; 13 Department of Breast and Thyroid Surgery, Yokohama City University Medical Center, Yokohama, Japan; 14 Department of Breast Surgery, Yokohama Minato Red Cross Hospital, Yokohama, Japan; 15 Department of Breast and Endocrine Surgery, St. Marianna University School of Medicine Hospital, Kawasaki, Japan; 16 Department of Surgery, Yong Loo Lin School of Medicine, National University of Singapore and National University Health System, Singapore; 17 Department of Hematology Oncology, National University Cancer Institute, National University Health System, Singapore; Mayo Clinic Arizona, UNITED STATES

## Abstract

**Purpose:**

Although association studies of genetic variations with the clinical outcomes of breast cancer patients treated with tamoxifen have been reported, genetic factors which could determine individual response to tamoxifen are not fully clarified. We performed a genome-wide association study (GWAS) to identify novel genetic markers for response to tamoxifen.

**Experimental design:**

We prospectively collected 347 blood samples from patients with hormone receptor-positive and human epidermal growth factor receptor 2-negative, invasive breast cancer receiving preoperative tamoxifen monotherapy for 14 to 28 days. We used Ki-67 response in breast cancer tissues after preoperative short-term tamoxifen therapy as a surrogate marker for response to tamoxifen. We performed GWAS and genotype imputation using 275 patients, and an independent set of 72 patients was used for replication study.

**Results:**

The combined result of GWAS and the replication study, and subsequent imputation analysis indicated possible association of three loci with Ki-67 response after tamoxifen therapy (rs17198973 on chromosome 4q34.3, rs4577773 on 6q12, and rs7087428 on 10p13, *P*_combined_ = 5.69 x 10^−6^, 1.64 x 10^−5^, and 9.77 x 10^−6^, respectively). When patients were classified into three groups by the scoring system based on the genotypes of the three SNPs, patients with higher scores showed significantly higher after/before ratio of Ki-67 compared to those with lower scores (*P* = 1.8 x 10^−12^), suggesting the cumulative effect of the three SNPs.

**Conclusion:**

We identified three novel loci, which could be associated with clinical response to tamoxifen. These findings provide new insights into personalized hormonal therapy for the patients with breast cancer.

## Introduction

The clinical benefit of the antiestrogen agent “tamoxifen” for the treatment of estrogen receptor (ER)-positive breast cancers is well-known [1]. It is reported that five-year tamoxifen therapy could improve the risk of its relapse for 15 years, particularly for ER–positive invasive breast cancers in premenopausal women [2]. However, inter-individual differences in response to tamoxifen therapy have been reported and 30–50% of patients with adjuvant tamoxifen therapy suffer a relapse and die of the disease [3, 4]. Many studies have suggested that metabolites of tamoxifen, 4-hydroxytamoxifen and 4-hydroxy-N-desmethyltamoxifen (endoxifen) are the active therapeutic moieties of tamoxifen. These two metabolites have at least 100-fold greater affinity to ER and 30–100 greater potency in inhibiting estrogen-dependent cell growth compared with a parent compound, tamoxifen [5–7]. Inter-individual differences in the production of these active metabolites could affect variability in the response to tamoxifen. Cytochrome P450 2D6 (CYP2D6) has been reported to be a key enzyme for the production of the potent active metabolites of tamoxifen, "4-hydroxytamoxifen" and "endoxifen" [8]. Many studies reported that decreased or null-function alleles of *CYP2D6* were associated with poor clinical outcome in breast cancer patients treated with tamoxifen [9–13]. However, the results showing the lack of association between *CYP2D6* genotypes and tamoxifen efficacy have also been reported [14–19], although these studies have been criticized for multiple issues as the cause of false-negative results [20]. To clarify the clinical significance of *CYP2D6* allele in tamoxifen therapy, we recently carried out the prospective *CYP2D6*-tamoxifen study and reported the positive association between *CYP2D6* genotype and response to tamoxifen using Ki-67 change in breast cancer tissues after short-term preoperative tamoxifen therapy [21], which has been known as a promising surrogate marker for clinical response to tamoxifen [21–24].

In addition to *CYP2D6*-tamoxifen study, many candidate gene approaches have been carried out [12, 15, 16, 18, 19, 25, 26], however, associations of the candidate genes have not yet been sufficiently validated. Hence, it has been suggested that the other genetic factors which could influence the inter-individual differences in responsiveness to tamoxifen should exist, even if the effects of genetic polymorphisms of *CYP2D6* were considered. In this study, to identify novel responsible loci for the response to tamoxifen therapy, we carried out a genome-wide association study (GWAS) and identified novel three loci associated with Ki-67 change after short-term tamoxifen therapy in the breast cancer patients treated with tamoxifen.

## Materials and methods

### Patients

A total of 347 patients with primary breast cancer (including the 233 patients reported previously [21]) were recruited at Showa University, Nippon Medical School, Tokyo Medical University, Saitama Cancer Center, Hirosaki Municipal Hospital, Sapporo Medical University, Sapporo Breast Surgical Clinic, Nakagami Hospital, Sagara Hospital, Yokohama City University Medical Center, Yokohama Minato Red Cross Hospital, St. Marianna University School of Medicine Hospital, and National University Cancer Institute, Singapore. Of them, 275 patients who were recruited from July 2012 to July 2014 were used for a GWAS analysis, and the remaining 72 patients who were recruited from October 2014 to November 2015 were analyzed in a replication study. All patients were women who were pathologically diagnosed with ER-positive (> 10%), human epidermal growth factor receptor 2 (HER2)-negative, invasive breast cancer without metastasis. ER status was evaluated by immunohistochemistry, and HER2 negativity was defined as < 2+ immunohistochemical staining or 2+ immunohistochemical staining without gene amplification by FISH test as previously described [21]. All patients received 20 mg/day of tamoxifen for 14 to 28 days until the day before the radical operation for breast cancer. Core-needle biopsy samples for diagnosis of the primary tumor were obtained before the first dose of tamoxifen, and tumor tissues after tamoxifen treatment were obtained from surgical specimen. Formalin fixation of tissues and record of Ki-67 labeling index was performed as described previously [21]. International Union Against Cancer TNM classification was used to determine the tumor and nodal status. This study was approved by the Institutional Review Boards of the National Cancer Center (Tokyo, Japan) and each participating institution. Written informed consent was obtained from all patients.

### Genotyping and quality control

In this study, genomic DNA was extracted from peripheral blood using a Qiagen DNA extraction kit (Qiagen, Hilden, Germany). For the GWAS analysis, 275 patients with primary breast cancer were genotyped at RIKEN Center for Integrative Medical Sciences using the Illumina HumanOmniExpressExome-8 v1.2 (Illumina, San Diego, USA). We performed single nucleotide polymorphism (SNP) quality control by excluding SNPs that had a low genotyping rate < 98%, showed deviations from Hardy–Weinberg equilibrium (*P* ≤ 1.0 x 10^−6^) and SNPs with minor allele frequency of < 0.05. 519,335 SNPs in autosomal chromosomes passed the quality control filters. We utilized the identity-by-state method to evaluate cryptic relatedness for the samples included in this study. Additionally, we examined population stratification by principal component analysis (PCA) using the EIGENSTRAT software v2.0 (https://www.hsph.harvard.edu/alkes-price/software/). The PCA was performed by comparing the distribution of the sample populations with three reference populations from the HapMap database that included Europeans (represented by Caucasian from UTAH, CEU), Africans (represented by Yoruba from Ibadan, YRI) and East Asians (represented by Japanese from Tokyo, JPT, and Han Chinese from Beijing, CHB). The top two principal components were utilized to produce a scatter plot for the identification of outliers who did not belong to the Asian cluster. The PCA was performed on the basis of the genotype information from the samples included in this study. The quantile–quantile (Q–Q) plot was generated between observed *P* values of Kruskal Wallis test against expected *P* values and revealed no significant population stratification with genomic inflation factor (λ = 1.020) (S1 Fig). In the replication study, 72 were genotyped using the Illumina HumanOmniExpressExome-8 v1.2 (Illumina), TaqMan SNP genotyping assay (Thermo Fisher Scientific, MA, USA), and Sanger sequencing.

### Genotype imputation

Genome-wide imputation was conducted separately for the 275 GWAS subjects. The reference panel that we used for imputation was based on the 1000 Genomes Project Phase 3 integrated release version 5 for individuals of East Asian descent comprising Japanese from Tokyo, Chinese from Beijing and Chinese from southern China. In brief, we first compared the allele frequencies in the GWAS and reference panels and exclude SNPs that have allele frequency dis-concordance at 0.15. We then phased the haplotypes for the samples using SHAPEIT ver2.0 software. Missing genotypes were imputed with Minimac3 software. We extracted variants with imputation quality (RSQ) threshold of RSQ > 0.9.

For imputation analysis, we performed SNP quality control by excluding SNPs that had a low genotyping rate < 98%, showed deviations from Hardy-Weinberg equilibrium (*P* ≤ 1.0 x 10^−6^) and SNPs with minor allele frequency of < 0.01.

### Statistical analysis

In the GWAS and the replication study, the differences in the Ki-67 labeling index among each genotype were evaluated by the Kruskal–Wallis test, and the Mann–Whitney *U* test was applied to two genetic models: a dominant-inheritance model, and a recessive-inheritance model. Significance levels after Bonferroni correction for multiple testing of three genetic models were *P* = 3.21 x 10^−8^ [0.05/(519,335x3)] in the GWAS stage and *P* = 2.03 x 10^−4^ [0.05/(82x3)] in replication analyses. For combination analysis, the genotype count of the replication study was added to that of the GWAS. For the predictive scoring system of Ki-67 response, we assigned a score of 2 to individuals homozygous for the risk allele (allele responsible for tamoxifen resistance) and 0 to individuals with the other genotypes at rs4577773 and rs7087428. We further assigned a scores of 2 and 1 to individuals homozygous and heterozygous for risk allele, respectively, and 0 to those without risk allele at rs17198973, and summed up the scores for each gene to obtain individuals’ scores. Based on this system, each patient was classified into any of the three prediction score groups (group 0, 1, ≥ 2). All the statistical analyses were carried out using R statistical environment version 3.3.1 (http://www.r-project.org/), PLINK version 1.07 [27], or the Ekuseru-Toukei 2015 (Social Survey Research Information Co., Ltd., Tokyo, Japan). Regional association plots were generated using Locus Zoom (http://locuszoom.sph.umich.edu/).

## Results

### Patient characteristics

We recruited 347 patients receiving short-term (14–28 days) preoperative tamoxifen monotherapy to evaluate the effect of tamoxifen on change of Ki-67 labeling index in breast cancer tissues. The characteristics of these 347 patients who were pathologically diagnosed to have an ER-positive, HER2-negative, invasive breast cancer were summarized in Table 1. Their median age at the time of surgery was 54 years old (range, 25–85 years). Among the characteristics in Table 1, none of them showed significant association with Ki-67 response after preoperative tamoxifen therapy in logistic regression analysis (S1 Table).

**Table 1:** Patient demographics and clinical characteristics.

Characteristics		GWAS (*N* = 275)				Replication (*N* = 72)				Total (*N* = 347)			
		No. of patients (%)				No. of patients (%)				No. of patients (%)			
Age at registration, years													
	Median	54				54.5				54			
	Range	25–85				31–79				25–85			
Menopausal status													
	Premenopausal	123	(	44.7	)	30	(	41.7	)	153	(	44.1	)
	Postmenopausal	150	(	54.5	)	41	(	56.9	)	191	(	55.0	)
	Unknown	2	(	0.7	)	1	(	1.4	)	3	(	0.9	)
Tumor size, cm													
	≤2	165	(	60.0	)	45	(	62.5	)	210	(	60.5	)
	>2	102	(	37.1	)	25	(	34.7	)	127	(	36.6	)
	Unknown	8	(	2.9	)	2	(	2.8	)	10	(	2.9	)
Nodal Status													
	Negative	189	(	68.7	)	52	(	72.2	)	241	(	69.5	)
	Positive	77	(	28.0	)	19	(	26.4	)	96	(	27.7	)
	Unknown	9	(	3.3	)	1	(	1.4	)	10	(	2.9	)
ER status													
	<1%	0	(	0.0	)	0	(	0.0	)	0	(	0.0	)
	1%-10%	0	(	0.0	)	0	(	0.0	)	0	(	0.0	)
	10%-33%	2	(	0.7	)	0	(	0.0	)	2	(	0.6	)
	33%-67%	7	(	2.5	)	3	(	4.2	)	10	(	2.9	)
	>67%	260	(	94.5	)	68	(	94.4	)	328	(	94.5	)
	≥10%, details unknown	6	(	2.2	)	1	(	1.4	)	7	(	2.0	)
Allred score (ER)^a^													
	<8	19	(	6.9	)	3	(	4.2	)	22	(	6.3	)
	8	148	(	53.8	)	30	(	41.7	)	178	(	51.3	)
	Unknown	108	(	39.3	)	39	(	54.2	)	147	(	42.4	)
PR status													
	<1%	31	(	11.3	)	6	(	8.3	)	37	(	10.7	)
	1%-10%	20	(	7.3	)	2	(	2.8	)	22	(	6.3	)
	10%-33%	30	(	10.9	)	9	(	12.5	)	39	(	11.2	)
	33%-67%	32	(	11.6	)	6	(	8.3	)	38	(	11.0	)
	>67%	158	(	57.5	)	48	(	66.7	)	206	(	59.4	)
	Unknown	4	(	1.5	)	1	(	1.4	)	5	(	1.4	)
Her-2													
	Negative	90	(	32.7	)	26	(	36.1	)	116	(	33.4	)
	1+	149	(	54.2	)	29	(	40.3	)	178	(	51.3	)
	2+(without amplification)	35	(	12.7	)	17	(	23.6	)	52	(	15.0	)
	Unknown	1	(	0.4	)	0	(	0.0	)	1	(	0.3	)

^a^ Composite of the percentage of cells that stained (scored on a scale of 0–5) and the intensity of their staining (scored on a scale of 0–3).

### GWAS and replication analysis

**Fig 1:**
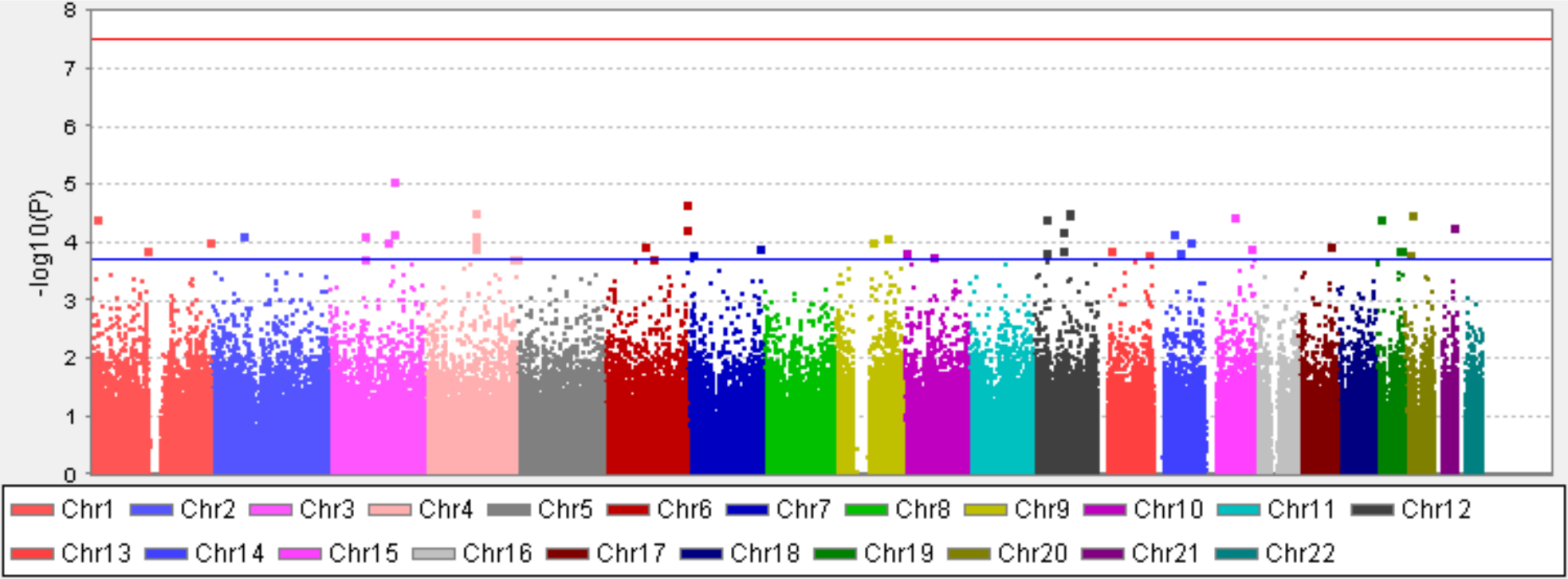
Manhattan plot of the GWAS. Manhattan plot showing the -log10-transformed *P* value of SNPs in the GWAS for Ki-67 response in 275 patients with breast cancer receiving preoperative tamoxifen monotherapy. The red line indicates the significance level in the GWAS (*P* < 3.21 x 10^−8^). The blue line indicates the threshold for *P* = 2.03 x 10^−4^ (Top 100 signals).

We used Ki-67 response after preoperative short-term tamoxifen therapy (after/before ratio of the Ki-67 index; when it is below 1, the proportion of Ki-67–positive cells is decreased) as a surrogate biomarker of tamoxifen efficacy because a change in the expression of Ki-67 after short-term tamoxifen therapy could be significantly associated with clinical response to tamoxifen [21–24]. To identify novel biomarker(s) for predicting response to tamoxifen therapy, we carried out a GWAS of Ki-67 response after tamoxifen therapy of 275 patients with breast cancer who received preoperative tamoxifen monotherapy using HumanOmniExpressExome-8 v1.2. The association analysis was carried out for 519,335 SNPs by the Kruskal-Wallis test and Mann–Whitney *U* test after the standard quality control. We generated a quantile-quantile plot to inspect possible population stratification effects and obtained the genomic inflation factor (λ_GC_) of 1.02, indicating no population substructure (S1 Fig). However, we could not observe the SNP which reach genome-wide significance level (Fig 1). The top 100 markers are displayed in S2 Table.

To further validate the results of the GWAS, we performed a replication study using an independent set of 72 hormone receptor-positive breast cancer patients receiving preoperative tamoxifen monotherapy. We genotyped the top 100 SNPs that had the most significant *P* values in the GWAS (S2 Table). We set significance levels for three genetic models after the Bonferroni correction at *P* = 2.03 x 10^−4^ [0.05/(82x3)] in replication study because 18 SNPs were highly linked (r^2^>0.80) to the other SNPs. Although we could not find any SNPs, which revealed significant levels of association in the replication stage, we identified possibly associated SNPs on a locus, chromosome 4q34 (rs4861477, *P*_min_ = 3.65 x 10^−2^, Table 2). A combined result of GWAS and the replication studies reveled stronger association of three SNPs than those in GWAS results (rs4861477 on chromosome 4q34.3, *P*_combined_ = 8.34 x 10^−6^, rs4577773 on chromosome 6q12, *P*_combined_ = 1.64 x 10^−5^, and rs7893556 on chromosome 10p13, *P*_combined_ = 2.03 x 10^−5^, Table 2).

**Table 2:** Summary of the association results of GWAS and replication study.

SNP	Chr	Chr location^a^	Gene	Allele 1/2(risk)	Stage	Genotyped /imputed	Number of patients			Risk allele frequency	*P* value		
							11	12	22		Mann-Whitney *U*-test_11vs^b^	Mann-Whitney *U*-test_22vs^c^	Kruskal-Wallis test_11vs12vs22
rs4861477	4	182,411,580	No gene	C/A(C)	GWAS	Genotyped	6	69	200	0.15	2.12E-02	9.01E-05	1.78E-04
					replication		2	16	54	0.14	3.98E-02	3.75E-02	3.65E-02
					combined		8	85	254	0.15	2.53E-03	9.86E-06	8.34E-06
rs17198973	4	182,407,878	No gene	C/T(C)	GWAS	Imputed	30	133	112	0.35	9.69E-04	2.21E-04	7.26E-05
					replication		9	32	31	0.35	2.23E-01	1.03E-02	3.45E-02
					combined		39	165	143	0.35	1.28E-04	4.49E-05	5.69E-06
rs4577773	6	63,543,538	No gene	G/A(G)	GWAS	Genotyped	69	132	74	0.49	5.34E-05	3.54E-02	2.18E-04
					replication		19	35	18	0.51	1.15E-01	2.07E-01	2.15E-01
					combined		88	167	92	0.49	1.64E-05	1.52E-02	5.74E-05
rs7893556	10	16,771,273	*RSU1*	T/C(T)	GWAS	Genotyped	11	82	182	0.19	7.86E-05	3.02E-02	2.28E-04
					replication		3	17	52	0.16	1.05E-01	1.02E-01	1.38E-01
					combined		14	99	234	0.18	2.03E-05	6.04E-03	3.38E-05
rs7087428	10	16,754,492	*RSU1*	C/A(C)	GWAS	Imputed	12	77	186	0.18	3.64E-05	1.19E-02	8.47E-05
					replication		2	17	53	0.15	1.00E-01	7.68E-02	1.02E-01
					combined		14	94	239	0.18	9.77E-06	1.90E-03	1.03E-05

Chr, chromosome; GWAS, genome-wide association study

^a^Based on GRCh 37 genome assembly

^b^11vs: Dominant or recessive-inheritance model of Mann Whitney U-test, depending on inheritance mode of allele 1.

^c^22vs: Dominant or recessive-inheritance model of Mann Whitney U-test, depending on inheritance mode of allele 2.

### Imputation analysis

**Fig 2:**
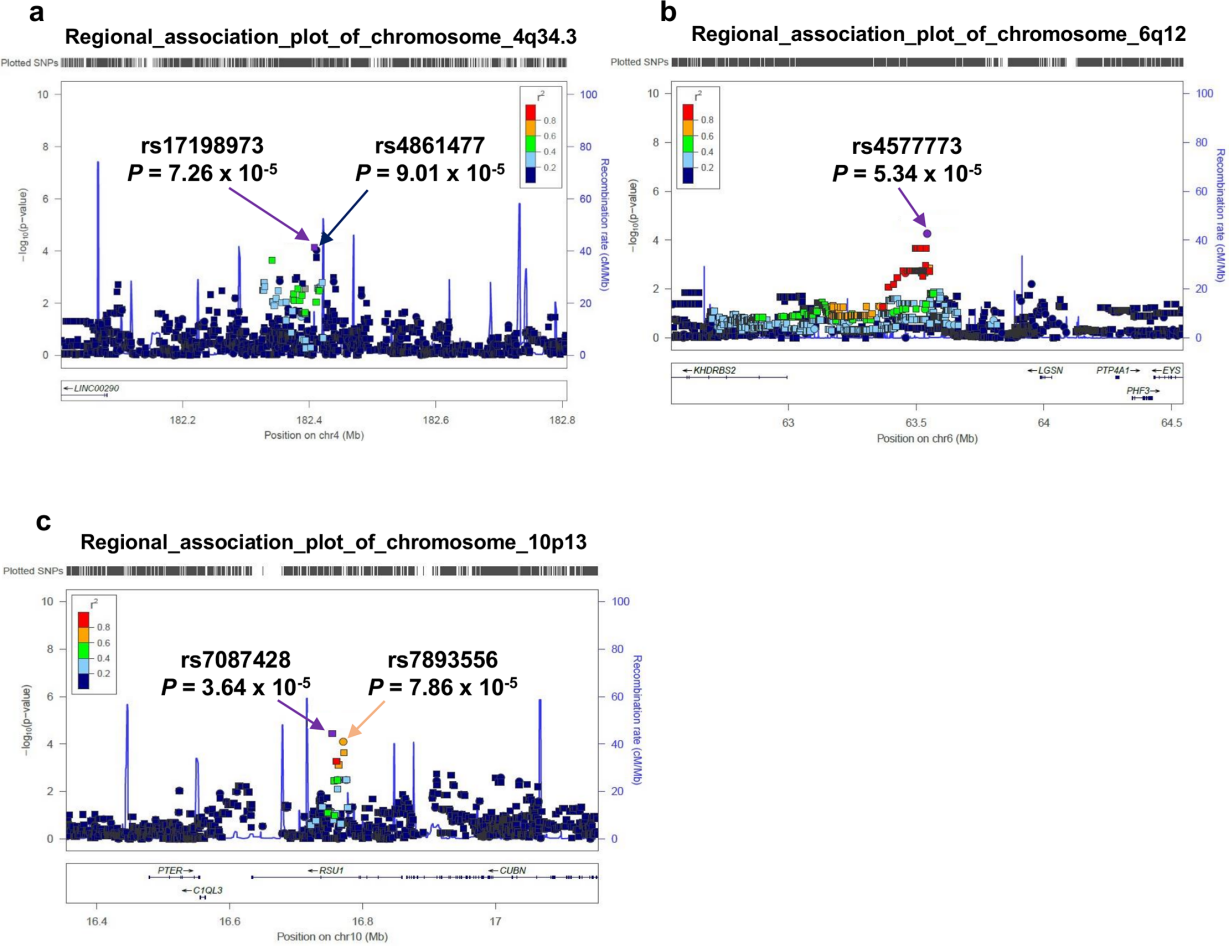
Regional association plots for three loci possibly associated with Ki-67 response after preoperative tamoxifen therapy. Upper panel; *P* values of genotyped SNPs (circle) and imputed SNPs (square) are plotted (as −log_10_*P* value) against their physical location on chromosome 4q34.3 (a), 6q12 (b) and 10p13 (c). The genetic recombination rates estimated from 1000 Genomes samples (JPT + CHB) are shown with a blue line. SNP's color indicates LD with rs17198973 (a), rs4577773 (b) and rs7087428 (c) according to a scale from *r*^2^ = 0 to 1 based on pair-wise *r*^2^ values from 1000 Genomes ASN. Lower panel; gene annotations from the University of California Santa Cruz genome browser.

To identify additional candidate loci associated with Ki-67 response after tamoxifen therapy, we examined associations by using genome-wide imputed genotypes of GWAS samples. Although this analysis identified additional 231 candidate variants (*P* < 1 x 10^−4^), we could not observe novel candidate locus associated with Ki-67 response. Of the above three candidate locus identified in GWAS (chromosome 4q34.3, 6q12 and 10p13), imputation analysis identified two novel SNPs on chromosome 4q34.3 and 10p13 which showed greater significance than the marker SNPs (rs17198973 on chromosome 4q34.3; *P* = 7.26 x 10^−5^, rs7087428 on chromosome 10p13; *P* = 3.64 x 10^−5^, Table 2, Fig 2). We further performed a replication study for the two SNPs, and found that the associations of the two imputed SNPs were stronger than those of the marker SNPs (rs17198973 on chromosome 4q34.3, *P* = 1.03 x 10^−2^ and rs7087428 on chromosome 10p13, *P* = 7.68 x 10^−2^, Table 2). A combined results of the GWAS and replication stage revealed stronger association of the two imputed SNPs with Ki-67 response than those of GWAS stage (rs17198973; *P*_combined_ = 5.69 x 10^−6^, and rs7087428; *P*_combined_ = 9.77 x 10^−6^, Table 2).

### Combination analysis of the three candidate SNPs associated with Ki-67 response after preoperative tamoxifen therapy

**Fig 3:**
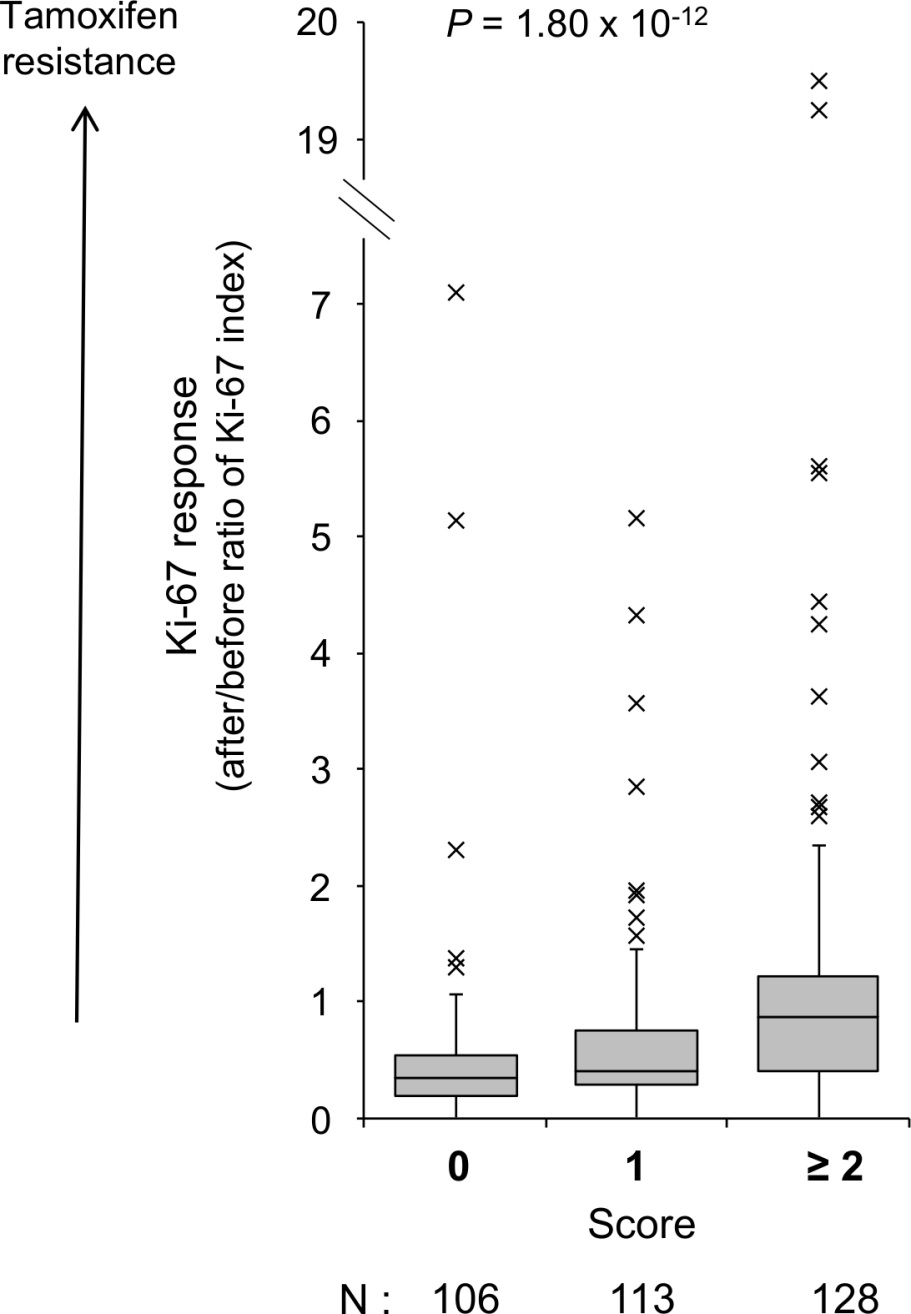
The relationship between Ki-67 response and the prediction score. Prediction score was significantly associated with the Ki-67 response in breast cancer tissues after tamoxifen therapy (*P* = 1.80 x 10^−12^).

We investigated combined effects of the three loci on the Ki-67 response after preoperative tamoxifen therapy using a scoring system because the three SNPs, which showed smallest *P* values in combined study at each locus (rs17198973, rs4577773 and rs7087428), were independent markers for Ki-67 response when analyzed by multivariate analysis (*P* < 7.18 x 10^−3^, S1 Table). For the predictive scoring system, each patient was scored considering the number of risk alleles (alleles responsible for increase of after/before ratio of the Ki-67, i.e., tamoxifen resistance alleles) of the three SNP; we gave a score of 2 to individuals homozygous for the risk allele, and 0 to those with other genotypes at rs4577773 and rs7087428 because the recessive-inheritance model revealed a smallest *P* value at the two SNPs. Furthermore, we gave scores of 2 and 1 to individuals homozygous and heterozygous for risk allele, respectively, and 0 to those without risk allele at rs17198973 because the additive model revealed a smallest *P* value at this SNP. Individuals’ scores were obtained by summing up the scores for each gene. We investigated a combined effect of the three SNPs on the Ki-67 response after preoperative tamoxifen therapy by classifying the 347 patients into 3 groups (0, 1, and ≥2 prediction score groups) according to the above scoring method (Fig 3). The patients with higher prediction scores indicated significantly higher after/before ratio of the Ki-67 index (more resistant to tamoxifen), suggesting that the predictive scoring system using the above three SNPs could predict the clinical response to tamoxifen (*P* = 1.80 x 10^−12^, Fig 3).

## Discussion

The use of effective drugs such as tamoxifen at the lower cost based on individual germline and/or somatic genetic information is important to reduce the medical cost with maintaining the quality of medical care for patients with ER-positive breast cancer. This study is the first prospective GWAS which attempts to identify genetic variants associated with clinical response to tamoxifen using Ki-67 response as a surrogate marker of its efficacy. Although the genome-wide significant SNPs was not identified in this study, we identified three candidate SNPs showing possible association with response to tamoxifen in three independent loci (Table 2). Moreover, predictive scoring system using the three candidate SNPs showed significantly higher after/before ratio of the Ki-67 index (more resistant to tamoxifen) in patients with higher score groups (higher ratio in score 2 or more >1 > 0 groups, *P* = 1.80 x 10^−12^, Fig 3). We further investigated the relationship between the prediction score and clinical outcome of breast cancer patients treated with tamoxifen in adjuvant setting, who were used in our previous retrospective study [26], and observed significant association of the prediction score with recurrence-free survival after tamoxifen therapy (Log-rank *P* = 0.0056, S2 Fig). These lines of evidence suggest the potential to improve the ability of physicians to select optimal hormonal drug based on the result of this predictive scoring system for the treatment of patients with hormone receptor–positive breast cancer.

The most strongly associated SNP in this study, rs17198973 (*P*_combined_ = 5.69 x 10^−6^) which was identified by genome-wide imputation, is located in the region on chromosome 4q34.3, although no protein-coding gene in this region is known. A GWAS reported that rs17198973 was one of the variants which could be associated with height [28]. Although body height could be regulated by genetic and environmental factors, plasma estrogen (estradiol) level has been reported as one of the factors which could influence the body height [29]. The interindividual variation of estrogen level could affect the response to tamoxifen because this drug acts by competitive antagonism of estrogen at its receptor site [30, 31]. Hence, rs17198973 might be one of the marker SNPs for response to tamoxifen, although further validation studies will be needed to prove the true association.

The second strongest association with Ki-67 response after preoperative tamoxifen therapy in this study was observed at rs7087428 (*P*_combined_ = 9.77 x 10^−6^) on chromosome 10p13 (Table 2). rs7087428 located in intron 7 of Ras Suppressor Protein 1 (*RSU1*). RSU1 is reported to be involved in the Ras signal effects in breast cancer by inhibiting anchorage-independent cancer cell proliferation [32–35]. Activation of Ras/Raf-1/MAPK is related to tamoxifen resistance through phosphorylation of estrogen receptor alpha [36–38]. Ras signal transduction could influence response to tamoxifen, and increase risk of relapse and death after tamoxifen treatment [39–42]. Therefore, genetic variations in *RSU1* might contribute to response to tamoxifen through effects on Ras/Raf-1/MAPK signaling.

Genetic variants of *CYP2C19*, *CYP3A5*, *UGT2B15* and *SULT1A1* have been investigated as candidate genes associated with clinical response to tamoxifen [12, 18, 25]. In our GWAS, the SNPs in these candidate genes showed no or weak association with Ki-67 response after preoperative tamoxifen therapy (*P* = 2.12 x 10^−2^–9.96 x 10^−1^). Moreover, the *P* values of the SNPs in the *ESR1*, *ESR2* and *PGR* genes, which encode ER-alpha, ER-beta and progesterone receptor (PR), respectively, were from 4.12 x 10^−2^ to 9.96 x 10^−1^, indicating no or weak association [43]. The sample size used in this study might not be enough to detect significant associations of these SNPs because the effect sizes of these candidate genes were not so large according to the previous reports [13, 18, 44].

In conclusion, our prospective GWAS using 347 patients with breast cancer identified three novel candidate loci, chromosome 4q34.3, 6q12, and 10p13, which were associated with Ki-67 response after preoperative tamoxifen therapy. Moreover, the combined analysis of the three SNP loci revealed that the predictive score based on risk genotypes was significantly associated with Ki-67 response after tamoxifen therapy. Our findings provide new insights into individualization of hormonal therapy for the patients with breast cancer. However, larger replication study and further functional analysis for candidate loci are required to verify these results and to clarify biological mechanisms which have effects on the clinical response to tamoxifen treatment.

## Supporting information

S1 Fig QQ plot in the GWAS. Vertical and horizontal lines represent expected p values under a null distribution and observed p values, respectively. If all the SNPs were not associated with the disease, all plots would lie on the line y = x.
(TIF)

S2 Fig The association between prediction scores and recurrence-free survival after adjuvant tamoxifen therapy. Patients with higher prediction scores (>2) in this study showed significantly shorter recurrence-free survival after adjuvant tamoxifen therapy compared to those with lower prediction scores (2 or less) in retrospective cohort used in our previous study [26].
(TIF)

S1 Table Logistic regression analysis for Ki-67 response. (XLSX)

S2 Table Association results of GWAS for the top 100 SNPs which revealed smallest P-value. (XLSX)

## References

[1] Li JJ, Shao ZM. Endocrine therapy as adjuvant or neoadjuvant therapy for breast cancer: selecting the best agents, the timing and duration of treatment. Chin Clin Oncol. 2016;5(3):40 doi: 10.21037/cco.2016.03.24 27164856 10.21037/cco.2016.03.24

[2] Davies C, Godwin J, Gray R, Clarke M, Cutter D, Darby S, et al Relevance of breast cancer hormone receptors and other factors to the efficacy of adjuvant tamoxifen: patient-level meta-analysis of randomised trials. Lancet. 2011;378(9793):771–84. 10.1016/S0140-6736(11)60993-8 21802721 PMC3163848

[3] Tamoxifen for early breast cancer: an overview of the randomised trials. Early Breast Cancer Trialists' Collaborative Group. Lancet. 1998;351(9114):1451–67. 9605801

[4] Effects of chemotherapy and hormonal therapy for early breast cancer on recurrence and 15-year survival: an overview of the randomised trials. Lancet. 2005;365(9472):1687–717. 10.1016/S0140-6736(05)66544-0 15894097

[5] Borgna JL, Rochefort H. Hydroxylated metabolites of tamoxifen are formed in vivo and bound to estrogen receptor in target tissues. J Biol Chem. 1981;256(2):859–68. 7451477

[6] Johnson MD, Zuo H, Lee KH, Trebley JP, Rae JM, Weatherman RV, et al Pharmacological characterization of 4-hydroxy-N-desmethyl tamoxifen, a novel active metabolite of tamoxifen. Breast Cancer Res Treat. 2004;85(2):151–9. 10.1023/B:BREA.0000025406.31193.e8 15111773

[7] Lien EA, Solheim E, Lea OA, Lundgren S, Kvinnsland S, Ueland PM. Distribution of 4-hydroxy-N-desmethyltamoxifen and other tamoxifen metabolites in human biological fluids during tamoxifen treatment. Cancer Res. 1989;49(8):2175–83. 2702659

[8] Desta Z, Ward BA, Soukhova NV, Flockhart DA. Comprehensive evaluation of tamoxifen sequential biotransformation by the human cytochrome P450 system in vitro: prominent roles for CYP3A and CYP2D6. J Pharmacol Exp Ther. 2004;310(3):1062–75. 10.1124/jpet.104.065607 15159443

[9] Goetz MP, Rae JM, Suman VJ, Safgren SL, Ames MM, Visscher DW, et al Pharmacogenetics of tamoxifen biotransformation is associated with clinical outcomes of efficacy and hot flashes. J Clin Oncol. 2005;23(36):9312–8. 10.1200/JCO.2005.03.3266 16361630

[10] Newman WG, Hadfield KD, Latif A, Roberts SA, Shenton A, McHague C, et al Impaired tamoxifen metabolism reduces survival in familial breast cancer patients. Clin Cancer Res. 2008;14(18):5913–8. 10.1158/1078-0432.CCR-07-5235 18794105

[11] Ramon y Cajal T, Altes A, Pare L, del Rio E, Alonso C, Barnadas A, et al Impact of CYP2D6 polymorphisms in tamoxifen adjuvant breast cancer treatment. Breast Cancer Res Treat. 2010;119(1):33–8. 10.1007/s10549-009-0328-y 19189210

[12] Schroth W, Antoniadou L, Fritz P, Schwab M, Muerdter T, Zanger UM, et al Breast cancer treatment outcome with adjuvant tamoxifen relative to patient CYP2D6 and CYP2C19 genotypes. J Clin Oncol. 2007;25(33):5187–93. 10.1200/JCO.2007.12.2705 18024866

[13] Schroth W, Goetz MP, Hamann U, Fasching PA, Schmidt M, Winter S, et al Association between CYP2D6 polymorphisms and outcomes among women with early stage breast cancer treated with tamoxifen. JAMA. 2009;302(13):1429–36. 10.1001/jama.2009.1420 19809024 PMC3909953

[14] Abraham JE, Maranian MJ, Driver KE, Platte R, Kalmyrzaev B, Baynes C, et al CYP2D6 gene variants: association with breast cancer specific survival in a cohort of breast cancer patients from the United Kingdom treated with adjuvant tamoxifen. Breast Cancer Res. 2010;12(4):R64 10.1186/bcr2629 20731819 PMC2949659

[15] Nowell SA, Ahn J, Rae JM, Scheys JO, Trovato A, Sweeney C, et al Association of genetic variation in tamoxifen-metabolizing enzymes with overall survival and recurrence of disease in breast cancer patients. Breast Cancer Res Treat. 2005;91(3):249–58. 10.1007/s10549-004-7751-x 15952058

[16] Okishiro M, Taguchi T, Jin Kim S, Shimazu K, Tamaki Y, Noguchi S. Genetic polymorphisms of CYP2D6 10 and CYP2C19 2, 3 are not associated with prognosis, endometrial thickness, or bone mineral density in Japanese breast cancer patients treated with adjuvant tamoxifen. Cancer. 2009;115(5):952–61. 10.1002/cncr.24111 19156902

[17] Regan MM, Leyland-Jones B, Bouzyk M, Pagani O, Tang W, Kammler R, et al CYP2D6 genotype and tamoxifen response in postmenopausal women with endocrine-responsive breast cancer: the breast international group 1–98 trial. J Natl Cancer Inst. 2012;104(6):441–51. 10.1093/jnci/djs125 22395644 PMC3309132

[18] Wegman P, Elingarami S, Carstensen J, Stal O, Nordenskjold B, Wingren S. Genetic variants of CYP3A5, CYP2D6, SULT1A1, UGT2B15 and tamoxifen response in postmenopausal patients with breast cancer. Breast Cancer Res. 2007;9(1):R7 10.1186/bcr1640 17244352 PMC1851378

[19] Wegman P, Vainikka L, Stal O, Nordenskjold B, Skoog L, Rutqvist LE, et al Genotype of metabolic enzymes and the benefit of tamoxifen in postmenopausal breast cancer patients. Breast Cancer Res. 2005;7(3):R284–90. 10.1186/bcr993 15987423 PMC1143572

[20] Kiyotani K, Mushiroda T, Zembutsu H, Nakamura Y. Important and critical scientific aspects in pharmacogenomics analysis: lessons from controversial results of tamoxifen and CYP2D6 studies. J Hum Genet. 2013;58(6):327–33. 10.1038/jhg.2013.39 23657426

[21] Zembutsu H, Nakamura S, Akashi-Tanaka S, Kuwayama T, Watanabe C, Takamaru T, et al Significant Effect of Polymorphisms in CYP2D6 on Response to Tamoxifen Therapy for Breast Cancer: A Prospective Multicenter Study. Clin Cancer Res. 2017;23(8):2019–26. 10.1158/1078-0432.CCR-16-1779 27797974

[22] DeCensi A, Guerrieri-Gonzaga A, Gandini S, Serrano D, Cazzaniga M, Mora S, et al Prognostic significance of Ki-67 labeling index after short-term presurgical tamoxifen in women with ER-positive breast cancer. Ann Oncol. 2011;22(3):582–7. 10.1093/annonc/mdq427 20716629

[23] Dowsett M, Smith IE, Ebbs SR, Dixon JM, Skene A, A'Hern R, et al Prognostic value of Ki67 expression after short-term presurgical endocrine therapy for primary breast cancer. J Natl Cancer Inst. 2007;99(2):167–70. 10.1093/jnci/djk020 17228000

[24] Viale G, Giobbie-Hurder A, Regan MM, Coates AS, Mastropasqua MG, Dell'Orto P, et al Prognostic and predictive value of centrally reviewed Ki-67 labeling index in postmenopausal women with endocrine-responsive breast cancer: results from Breast International Group Trial 1–98 comparing adjuvant tamoxifen with letrozole. J Clin Oncol. 2008;26(34):5569–75. 10.1200/JCO.2008.17.0829 18981464 PMC2651094

[25] Gjerde J, Geisler J, Lundgren S, Ekse D, Varhaug JE, Mellgren G, et al Associations between tamoxifen, estrogens, and FSH serum levels during steady state tamoxifen treatment of postmenopausal women with breast cancer. BMC cancer. 2010;10:313 10.1186/1471-2407-10-313 20565970 PMC2910688

[26] Kiyotani K, Mushiroda T, Imamura CK, Hosono N, Tsunoda T, Kubo M, et al Significant effect of polymorphisms in CYP2D6 and ABCC2 on clinical outcomes of adjuvant tamoxifen therapy for breast cancer patients. J Clin Oncol. 2010;28(8):1287–93. 10.1200/JCO.2009.25.7246 20124171 PMC4872305

[27] Purcell S, Neale B, Todd-Brown K, Thomas L, Ferreira MA, Bender D, et al PLINK: a tool set for whole-genome association and population-based linkage analyses. Am J Hum Genet. 2007;81(3):559–75. 10.1086/519795 17701901 PMC1950838

[28] Kent JW Jr., Peterson CP, Dyer TD, Almasy L, Blangero J. Genome-wide discovery of maternal effect variants. BMC Proc. 2009;3 Suppl 7:S19. 20018008 10.1186/1753-6561-3-s7-s19 PMC2795915

[29] Schuit SC, van Meurs JB, Bergink AP, van der Klift M, Fang Y, Leusink G, et al Height in pre- and postmenopausal women is influenced by estrogen receptor alpha gene polymorphisms. J Clin Endocrinol Metab. 2004;89(1):303–9. 10.1210/jc.2003-031095 14715865

[30] Criscitiello C, Fumagalli D, Saini KS, Loi S. Tamoxifen in early-stage estrogen receptor-positive breast cancer: overview of clinical use and molecular biomarkers for patient selection. Onco Targets Ther. 2010;4:1–11. 10.2147/OTT.S10155 21552410 PMC3084302

[31] Rondon-Lagos M, Rangel N, Di Cantogno LV, Annaratone L, Castellano I, Russo R, et al Effect of low doses of estradiol and tamoxifen on breast cancer cell karyotypes. Endocr Relat Cancer. 2016;23(8):635–50. 10.1530/ERC-16-0078 27357940 PMC5064758

[32] Cutler ML, Bassin RH, Zanoni L, Talbot N. Isolation of rsp-1, a novel cDNA capable of suppressing v-Ras transformation. Mol Cell Biol. 1992;12(9):3750–6. 1508180 10.1128/mcb.12.9.3750-3756.1992 PMC360236

[33] Dougherty GW, Jose C, Gimona M, Cutler ML. The Rsu-1-PINCH1-ILK complex is regulated by Ras activation in tumor cells. Eur J Cell Biol. 2008;87(8–9):721–34. 10.1016/j.ejcb.2008.02.011 18436335 PMC2600675

[34] Giotopoulou N, Valiakou V, Papanikolaou V, Dubos S, Athanassiou E, Tsezou A, et al Ras suppressor-1 promotes apoptosis in breast cancer cells by inhibiting PINCH-1 and activating p53-upregulated-modulator of apoptosis (PUMA); verification from metastatic breast cancer human samples. Clin Exp Metastasis. 2015;32(3):255–65. 10.1007/s10585-015-9701-x 25647720

[35] Gkretsi V, Stylianou A, Louca M, Stylianopoulos T. Identification of Ras suppressor-1 (RSU-1) as a potential breast cancer metastasis biomarker using a three-dimensional in vitro approach. Oncotarget. 2017;8(16):27364–79. doi: 10.18632/oncotarget.16062 28423706 10.18632/oncotarget.16062 PMC5432341

[36] Janes PW, Daly RJ, deFazio A, Sutherland RL. Activation of the Ras signalling pathway in human breast cancer cells overexpressing erbB-2. Oncogene. 1994;9(12):3601–8. 7970720

[37] Kato S, Endoh H, Masuhiro Y, Kitamoto T, Uchiyama S, Sasaki H, et al Activation of the estrogen receptor through phosphorylation by mitogen-activated protein kinase. Science. 1995;270(5241):1491–4. 7491495 10.1126/science.270.5241.1491

[38] Nicholson RI, McClelland RA, Robertson JF, Gee JM. Involvement of steroid hormone and growth factor cross-talk in endocrine response in breast cancer. Endocr Relat Cancer. 1999;6(3):373–87. 10516852 10.1677/erc.0.0060373

[39] Ali S, Coombes RC. Endocrine-responsive breast cancer and strategies for combating resistance. Nat Rev Cancer. 2002;2(2):101–12. 10.1038/nrc721 12635173

[40] Hurtado A, Holmes KA, Geistlinger TR, Hutcheson IR, Nicholson RI, Brown M, et al Regulation of ERBB2 by oestrogen receptor-PAX2 determines response to tamoxifen. Nature. 2008;456(7222):663–6. 10.1038/nature07483 19005469 PMC2920208

[41] Schiff R, Massarweh S, Shou J, Osborne CK. Breast cancer endocrine resistance: how growth factor signaling and estrogen receptor coregulators modulate response. Clin Cancer Res. 2003;9(1 Pt 2):447s–54s. 12538499

[42] Schiff R, Massarweh SA, Shou J, Bharwani L, Mohsin SK, Osborne CK. Cross-talk between estrogen receptor and growth factor pathways as a molecular target for overcoming endocrine resistance. Clin Cancer Res. 2004;10(1 Pt 2):331s–6s. 14734488 10.1158/1078-0432.ccr-031212

[43] Kuo SH, Yang SY, You SL, Lien HC, Lin CH, Lin PH, et al Polymorphisms of ESR1, UGT1A1, HCN1, MAP3K1 and CYP2B6 are associated with the prognosis of hormone receptor-positive early breast cancer. Oncotarget. 2017;8(13):20925–38. doi: 10.18632/oncotarget.14995 28178648 10.18632/oncotarget.14995 PMC5400556

[44] Kiyotani K, Mushiroda T, Tsunoda T, Morizono T, Hosono N, Kubo M, et al A genome-wide association study identifies locus at 10q22 associated with clinical outcomes of adjuvant tamoxifen therapy for breast cancer patients in Japanese. Hum Mol Genet. 2012;21(7):1665–72. 10.1093/hmg/ddr597 22180457

