# Supplementary figures and images for: A genome-wide association study identifies three novel genetic markers for response to tamoxifen: A prospective multicenter study
Source: PLoS One. 2018 Aug 30;13(8):e0201606. doi: 10.1371/journal.pone.0201606 (PMC6116947; doi:10.1371/journal.pone.0201606)

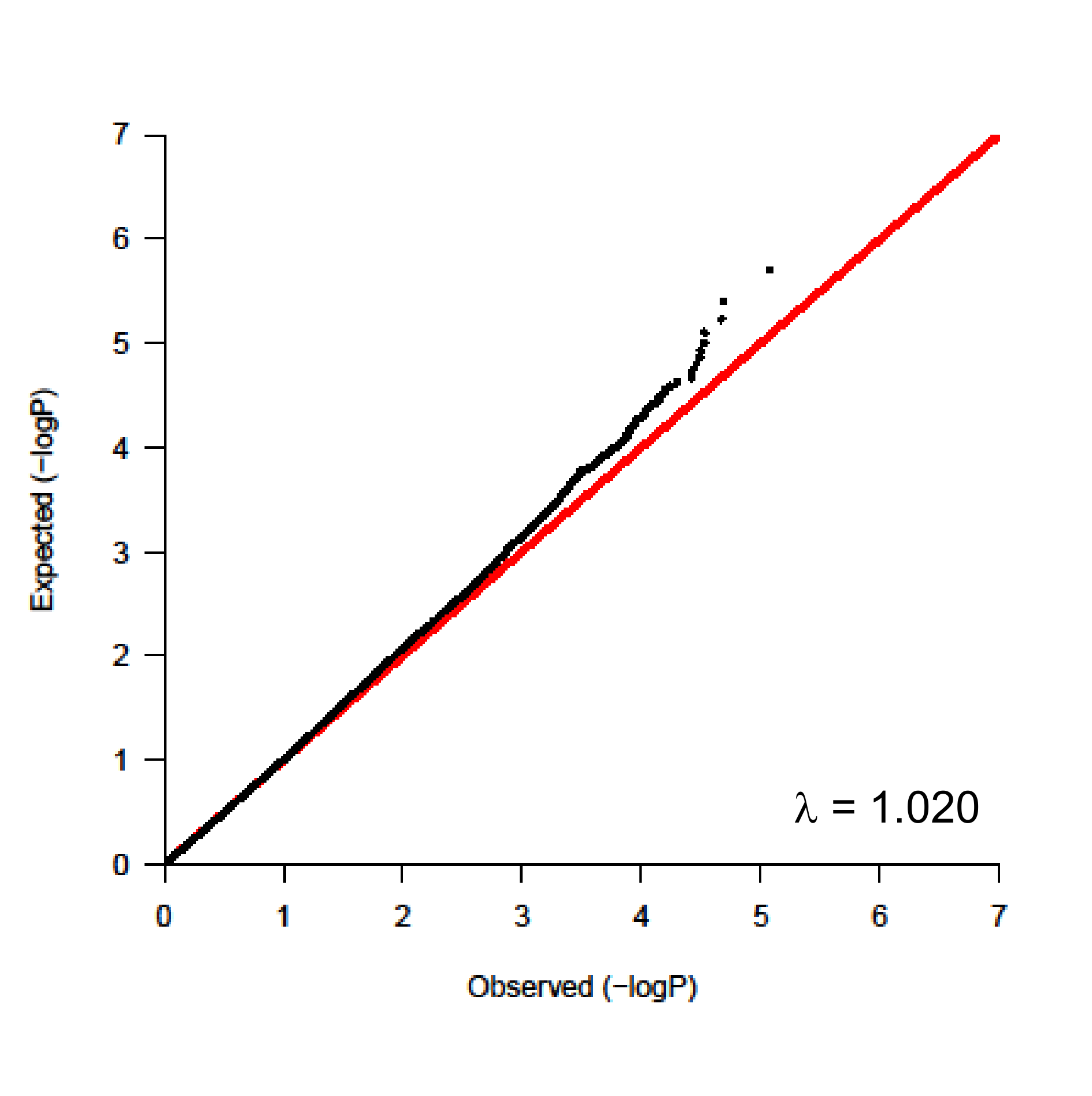

Supplement: S1 Fig — Vertical and horizontal lines represent expected p values under a null distribution and observed p values, respectively. If all the SNPs were not associated with the disease, all plots would lie on the line y = x. (TIF) [file pone.0201606.s001.tif]

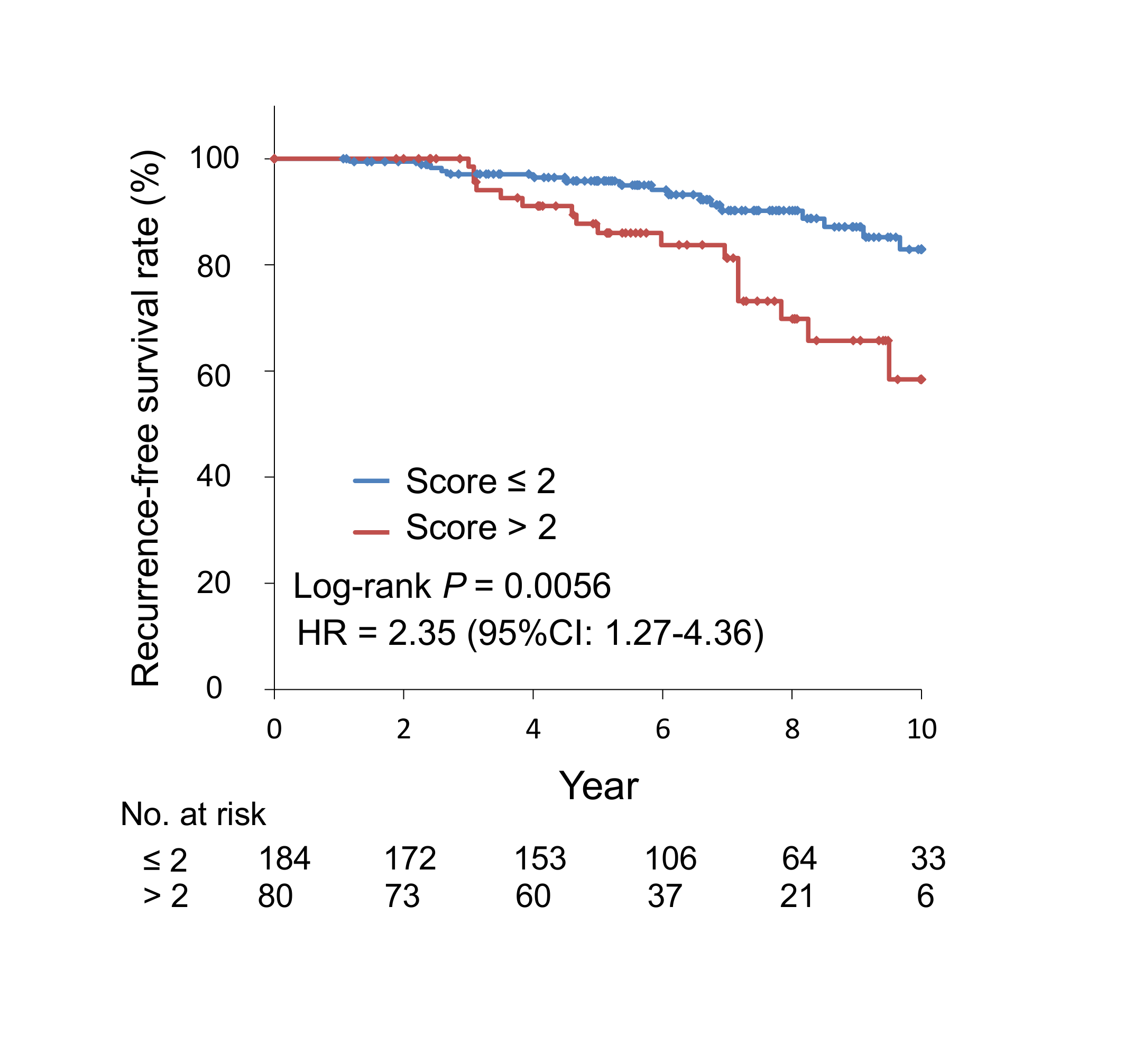

Supplement: S2 Fig — Patients with higher prediction scores (>2) in this study showed significantly shorter recurrence-free survival after adjuvant tamoxifen therapy compared to those with lower prediction scores (2 or less) in retrospective cohort used in our previous study [26]. (TIF) [file pone.0201606.s002.tif]
